# Relationship of Forced Social Distancing and Home Confinement Derived from the COVID-19 Pandemic with the Occupational Balance of the Spanish Population

**DOI:** 10.3390/jcm9113606

**Published:** 2020-11-09

**Authors:** Jerónimo J. González-Bernal, Mirian Santamaría-Peláez, Josefa González-Santos, Paula Rodríguez-Fernández, Benito León del Barco, Raúl Soto-Cámara

**Affiliations:** 1 Department of Health Sciences, University of Burgos, 09001 Burgos, Spain; jejavier@ubu.es (J.J.G.-B.); prf0011@alu.ubu.es (P.R.-F.); rscamara@ubu.es (R.S.-C.); 2 Department of Psychology, Faculty of Teacher Training College, University of Extremadura, 10071 Caceres, Spain; bleon@unex.es

**Keywords:** occupational balance, pandemic, COVID-19, social distancing, home confinement

## Abstract

Abrupt interruption in the performance of everyday occupations as a consequence of forced social distancing and home confinement, coupled with a lack of regulatory capacities and skills, can trigger harmful effects on people’s health and well-being. This study aimed to determine the factors related to the occupational balance in the Spanish population during home confinement as a consequence of the coronavirus disease 19 (COVID-19) pandemic. A total of 3261 subjects completed an online survey, which was disseminated through the mainstream social media platforms in Spain and included the Occupational Balance Questionnaire (OBQ), sociodemographic variables, and factors related to COVID-19 infection. The mean age of the participants (81.69% women) was 40.53 years (SD ± 14.05). Sociodemographic variables were related to a greater occupational balance, and the multivariate analysis showed that age (β = 0.071; *p* = 0.001), the perception of having received enough information (β = 0.071; *p* ≤ 0.001), not telecommuting (β = −0.047; *p* = 0.022), and not being infected by COVID-19 (β = 0.055; *p* = 0.007) contributed to a better occupational balance. There were profiles of people less likely to suffer disturbances in occupational balance during home confinement, but more studies are needed to help understand and analyze the effects of the COVID-19 pandemic on people’s occupational and mental health.

## 1. Introduction

Occupational balance is defined as the state in which there is a positive evaluation between the number of occupations and their variations [1]. Traditionally, and from an intrapersonal perspective, occupational balance has been described as a satisfactory pattern that the subject presents when carrying out their occupations [2,3,4]. Occupations are defined as all types of life activities in which people, groups, or populations participate, including activities of daily living, instrumental activities of daily living, rest and sleep, education, work, play, leisure, and socializing [5]. In this sense, the actions cited by Meyer [5]—work, play, rest, and sleep—should always be taken into account when considering what each person needs to achieve balance, even in difficult situations.

In order to maintain an occupational balance over time, there must be harmony and variation in occupations, capacity and resources to be able to manage them, and congruence between occupational commitment, values, and personal meaning [6]. However, despite the fact that an adequate occupational balance has been positively related to the health and well-being [7,8] of a person, recent research has highlighted the importance of the interpersonal perspective, which involves having to satisfy needs, such as family, or to avoid harming others [9,10,11].

Factors such as quality and satisfaction with life [7] and subjective health [12] can affect the achievement of this balance by hindering access to necessary resources. In this situation, states of underemployment or overoccupation may occur as a consequence of a deficient or exaggerated processing of occupational and environmental stimuli, of few or too many occupational opportunities, or of understimulating, overstimulating, or inappropriate environments [7]. It is therefore a dynamic process in which occupations, roles, personal factors, and external factors vary throughout the lifecycle, with the existence of a certain degree of disturbances in occupational balance being normal, as long as it is not too intense and durable over time and the person has the necessary capacities and resources to adapt and regain balance by readjusting their occupations [13].

A lack of regulatory capacities and abilities can be detrimental to a person’s health and lead to illness. Responses to the understimulating occupations or excess occupations, such as boredom or exhaustion, respectively, are the most frequent forms of stress when occupational balance is disturbed. These forms of stress are associated with a poor state of health and can lead to more severe medical conditions [14]. Based on all of the above, this disturbance is defined as any perception of decompensation in daily occupations, which translates into personal dissatisfaction and difficulty in adapting to particular circumstances [15]. It can be caused by both positive and negative situations [16]. In the former, it is the person himself who has chosen the disturbances directly, such as going to university or starting a new job. In the latter, the disturbances are not voluntarily chosen by the person, such as being unemployed, contracting a disease, or experiencing a global pandemic.

In the general population, the measures adopted by the Health Authorities to contain the spread of the coronavirus disease (COVID-19) outbreak, mainly focused on forced social distancing and home confinement, have produced negative psycho-emotional effects due to the loss of leisure and work, alterations in lifestyles, and the generation of stress [17]. The abrupt interruption in the performance of daily occupations has favored the adoption of health risk behaviors, such as physical inactivity, sedentary lifestyles, and excessive alcohol consumption [18], producing high levels of anxiety and depression [19,20,21]. All of this suggests that the lack of occupational balance translates into a general shortage of regulatory skills and capacities, with negative effects on health, which can lead to serious illnesses [22,23,24,25]. The feelings of loss of control and loneliness, caused by confinement and social distancing, have increased the incidence of daytime stress, altered sleep patterns [21,26,27,28], and increased irritability [21], as well as the almost-complete loss of structured occupations (school, work, and training) in young adults, one of the groups most affected by the effects of this situation [29,30]. In addition, adverse situations may encourage new lifestyle demands to exceed the individual’s capacities to face them, negatively affecting their health status [14].

The analysis of the impact of confinement on occupational balance is key to provide meaningful data that allow an approach to the situation focused on reducing or eliminating changes in behaviors and lifestyles produced by the pandemic, increasing quality of life, and reducing its effects on psycho-emotional health. We hypothesized that there was a relationship between sociodemographic and COVID-19-related factors and occupational balance in the Spanish population during the forced social distancing. Therefore, the objective of this study was to determine the factors related to occupational balance in the Spanish population during the confinement that occurred as a consequence of the COVID-19 outbreak.

## 2. Materials and Methods

### 2.1. Study Design—Participants

A cross-sectional study was designed. The study population consisted of people aged 18 years or above, who resided in Spain during the forced home confinement phase confinement that occurred as a consequence of the COVID-19 outbreak, and who had the necessary means and resources to access the study survey.

### 2.2. Procedure—Data Collection

The participants were selected by a non-probabilistic convenience sampling based on the voluntary character of the study. The data was obtained using an online survey through Google Forms, and the link was broadcasted through mainstream social media platforms, such as Facebook, Instagram, Twitter, and WhatsApp. This procedure represented the best data collection strategy in the phase of forced social distancing, allowing us to reach the largest number of people. The survey was available from 16 March to 10 May 2020, the time interval between the first working day of forced home confinement and the first working day on which the forced home confinement rules were relaxed. In the first part of the survey, a brief presentation informed the participants about the aims of the study, requesting their anonymous and voluntary participation. Participants could withdraw from the study at any time, without providing any justification, and the data were not saved. To guarantee anonymity, no personal data which could allow the identification of participants were collected. The completed return of the survey implied the electronic informed consent of the person to participate in the study. Only the questionnaire data that had a complete set of answers were considered. The required time to answer the survey was approximately 15–20 min.

The study received a favorable report from the Bioethics Committee of the Burgos University (Reference IR 14/2020) and was conducted in accordance with the ethical principles of the Declaration of Helsinki.

### 2.3. Main Outcomes—Instruments

Occupational balance was evaluated by the Occupational Balance Questionnaire (OBQ), designed by Wagman and Håkansson in 2014 [31] and validated for the Spanish population by Peral Gómez in 2017 [32]. This self-administered questionnaire includes 13 items and explores the balance between the different types of activities, their significance, time spent on each activity, and perceived satisfaction. Each item must be answered using a six-point Likert scale, according to the degree of agreement with the content of the sentence, where 0 corresponds to “completely disagree” and 5 to “totally agree.” The total score can range from 0 to 65, with higher scores indicating greater occupational balance [31]. Participants had to answer this questionnaire twice. In the first questionnaire, their responses had to refer to the week before the forced home confinement, while in the second one, responses referred to the time of the survey completion. The variation in occupational balance was the main outcome of the study, which was assessed by calculating the differential scores between each questionnaire.

Other variables were collected through an ad hoc questionnaire, previously piloted in a sample of 15 people, who were not part of the subsequent analysis. These variables were classified as sociodemographic (age, sex, marital status, employment status, educational level, home size and number of stays, number of people who make up the family nucleus, possibility of private access to the outside, setting), or COVID-19-related aspects (days of confinement at the time of completing the questionnaire, employment status, telecommuting, suffering or having suffered from COVID-19, experiencing required home isolation, perception of the information received).

### 2.4. Statistical Analysis

Descriptive analyses were conducted to describe sociodemographic characteristics, COVID-19-related aspects, and occupational balance. The categorical variables were expressed as absolute frequencies and percentages, while the continuous variables were expressed in terms of mean and standard deviation (SD). The compliance of the normality criteria of the quantitative variables was assessed by the Kolmogorov–Smirnov test. In cases where the normal distribution could not be assumed, the contributions made by Blanca et al. [33] were considered. To evaluate the association between the variation in the occupational balance and the categorical variables, the analysis of variance (ANOVA) and the post-hoc less significant differences (LSD) test were used. The effect size differences were calculated using partial eta squared (η^2^ *p*) or Hedge’s g and interpreted according to the following criteria: If 0 ≤ η^2^ *p* or *g* < 0.01, there is no effect; if 0.01 ≤ η^2^ *p* or g < 0.06, the effect is minimal; if 0.06 ≤ η^2^ *p* or g < 0.14, the effect is moderate; and if η^2^ *p* or g ≥ 0.14, the effect is strong. The relationship between the differential score obtained in the OBQ and the quantitative variables was analyzed using the Pearson correlation. A forward stepwise multiple lineal regression analysis, adjusted by sex and age, was performed to identify possible independent predictive factors, related to COVID-19 infection, for a higher occupational balance. In this model, variables with a *p*-value < 0.05 in the univariate analysis were included. Statistical analysis was performed with SPSS version 25 software (IBM-Inc, Chicago, IL, USA). For the analysis of statistical significance, a value of *p* < 0.05 was established.

## 3. Results

The study sample comprised 3261 subjects, with a majority of women over men (81.69% versus 18.31%). The age of the participants ranged from 18 to 93, with a mean of 40.53 years (SD ± 14.05). Of the 2412 participants who were previously working or studying, 66.92% (*n* = 1614) continued working or telecommute during the home confinement phase. At the time of completing the survey, 3.25% of participants reported that they had suffered or were currently suffering from COVID-19 infection. The perception of having received insufficient information about the infection by COVID-19 was generalized, with 2043 persons reporting it. The time elapsed between the beginning of home confinement and the filling of the questionnaire ranged between 4 and 57 days. The mean differential score obtained in the OBQ was (−10.45) (SD ± 18.25).

When comparing the differential scores of the occupational balance with the sociodemographic variables, marital status, educational level, employment status, private access to the outside, and housing size were related to disturbances in occupational balance (Table 1). Widowers were the group with the lowest occupational balance during home confinement when compared with married (*p* < 0.001) and single (*p* = 0.003) people. In terms of employment status, subjects with active employment status presented a greater occupational balance than students (*p* < 0.001), unemployed people (*p* = 0.001), and retired people (*p* < 0.001). Likewise, a higher occupational balance was observed in the students in relation to the unemployed (*p* = 0.05). A greater trend toward occupational balance was observed with higher levels of education and larger housing sizes. Private access to the outside showed a strong effect size on the differential scores of the occupational balance (*p* = 0.001; η^2^ *p* = 0.212), whereas the effect size of the employment status was minimal (*p* < 0.001; g = 0.14).

Likewise, the differential scores of occupational balance were weakly and positively correlated with age (*p* = 0.048) and the number of children under 18 years old at home (*p* = 0.003), and were weakly and negatively with the number of days in confinement (*p* < 0.001), with a greater mismatch over the days. At home, the number of people living together (*p* = 0.252), the number of dependents (*p* = 0.061), and the number of rooms (*p* = 0.232) did not show a significant relationship with the differential scores of occupational balance produced by confinement (Table 2).

The comparison of the differential scores of occupational balance according to the COVID-19-related variables showed a lower occupational balance in people who were not telecommuting previously (*p* = 0.033; g = 0.087), people who were infected by the virus (*p* = 0.004; g = 0.470) or isolated (*p* = 0.048; g = 0.165) at the time of filling in the form, and people who perceived the information received regarding the pandemic as insufficient (*p* < 0.001; g = 0.191) (Table 3). All of these factors showed a strong effect size on the differential scores of occupational balances except telecommuting, for which it was moderate.

Multivariate analysis showed that the major contributors to higher occupational balance during the confinement phase were age, the perception of having received enough information, not telecommuting, and not being infected by the COVID-19 at the time of filling out the form (Table 4). Other factors included in the regression model were not significantly associated with occupational balance.

## 4. Discussion

The measures adopted to contain the spread of the COVID-19 outbreak, based on forced social distancing and home confinement, were not significantly related to occupational balance in the Spanish population. A low educational level, being unemployed, being a widower or not having a partner, living in small house without private access to the outside, having a fewer number of children under 18 years old at home, being isolated, and spending a greater number of days in home confinement were related to a lower occupational balance. Regarding the variables related to COVID-19 infection, being older, the perception of having received enough information during the pandemic, not telecommuting, and not being infected at the time of filling out the form were independent predictive factors of a higher occupational balance.

People are born with an innate occupational nature, which drives them to occupy themselves and build their occupational identity. However, heterogeneity in what each person considers important makes it difficult to determine who is most vulnerable to suffering disturbances in occupational balance [34].

Despite being one of the first studies to describe the factors related to the occupational balance during home confinement, some research has already explored the differences between sociodemographic groups. Matuska et al. [13] observed that the profile of the people with a higher occupational balance was those 61 years or older, who had earned a master´s degree, and who had children under their care. Similar results were found in this study.

People with active employment, including those who went to the workplace in person and those who telecommuted from home, did not suffer a revealing disturbance in occupational balance compared to those who were unemployed, retired, or students. This result is consistent with previous research, in which financial security, provided by having a job, was a determining factor in achieving balance with occupations and with life [34,35].

The study by Wagman et al. [34], whose objective was to explore the factors that people consider to be more or less relevant to occupational balance, showed the importance of social relationships. A systematic review by Kamalakannan and Chakraborty [36] highlighted the limited attention paid to occupations during the COVID-19 outbreak compared to previous pandemics. In addition, these authors observed that people whose most significant occupations were related to social and leisure activities were most affected by home confinement. In this study, despite the lack of evidence of a significant relationship between the number of people living at home and occupational balance, widowers showed lower occupational balance than single people and people who lived with a partner, as well as those who were isolated in a single room at some point in home confinement. These findings reiterate the importance of social relationships in achieving occupational balance, highlighting the interpersonal perspective mentioned above [11].

There is a trend for a worse occupational balance as the days of home confinement progress, probably due to increased levels of stress, anxiety and depression [23]. Occupational balance is significantly and positively correlated with physical and mental health [6,22,34,37] and predicts the perceived stress by the general population [13], with a higher incidence in so-called risk groups [38].

In a recent study carried out in the Spanish population, it was shown that certain sociodemographic characteristics, such as being female, having minor children, and having a low educational level, increased the perception of threat of COVID-19 infection, facilitating the appearance of symptoms of anxiety or stress [38]. The results of another study, whose objective was to analyze the relation between the psychological impact of the pandemic and the national confinement experienced in Spain, concluded that excessive exposure to the mass media, living with individuals with chronical illness, and living with children under 12 years old increased fears of COVID-19 infection and its emotional consequences. However, an older age, a higher income level, working outside the home, having a private garden at home, and having positive affection have been considered protective factors [39]. The abovementioned results partially coincide with those obtained in this study, since factors such as age, the perception of having received enough information, working outside the home, and not being infected by COVID-19 at the time of filling out the form contributed to a better occupational balance during home confinement.

This study gives a picture of the occupational balance of the Spanish population during the COVID-19 pandemic. However, the study findings must be considered within the context of their limitations. Despite the large sample size, it was not possible to overcome the limitation of a cross-sectional study, and we were unable to determine a causal relationship between the variables. The use of an online survey presented a selection bias in participant recruitment, which was expressed by some characteristics of the sample, such as the high number of females. Measuring occupational balance on a single occasion, but referring to two different moments in time, may have influenced the responses given by the participants. However, using this data collection strategy made it possible to determine the occupational balance of the participants prior to the forced social distancing phase to avoid the participants´ loss during the follow-up period. The use of a convenience sampling may have induced the collection of responses primarily from people who felt strongly about the considered issue. Also, the lack of studies on this topic made it difficult to contrast the results obtained. These limitations can reduce the generalizability of the findings and may have influenced the results of the study. The strengths of this study include the collection of data on a large sample of Spanish adults and the analysis of a wide set of variables, including novel data such as length of confinement, telecommuting, and private access to the outside.

This type of research can promote social and health initiatives for the prevention and treatment of the possible effects of the pandemic and home confinement on the most vulnerable population, for example, by providing psychological and social support, promoting access to resources that reduce social isolation, or proposing measures to stop COVID-19 that take into account the needs of the population which affect their life balance.

Future studies are recommended to help understand and analyze the effects of the COVID-19 pandemic on people’s occupational and mental health.

## 5. Conclusions

The disturbances in occupational balance, caused by the home confinement derived from the COVID-19 pandemic, increased as the days progressed. Sociodemographic variables, such as marital status, educational level, employment situation, private access to the outside, and the size of the house, significantly affected the occupational balance. Specifically, married people, with an active work, a higher educational level, large houses, and with private access to the outside reported a better occupational balance during home confinement. Regarding the variables related to COVID-19, the main contributors to a greater occupational balance were age, the perception of having received enough information, working outside the home, and not currently being infected.

## Figures and Tables

**Table 1:** Comparison of differential scores in the OBQ according to sociodemographic variables.

	*n* (%)	Differential Score in OBQ		*p*-Value	Effect Size
		Mean	SD		
**Sex**					
Female	2664 (81.69)	(−10.30)	18.72	0.332	0.044 *
Male	597 (18.31)	(−11.10)	15.95		
**Marital status**					
Single/Separated—Divorced	1154 (35.39)	(−10.98)	18.93	0.002	0.004 **
Married—Living with a partner	2040 (62.56)	(−9.92)	17.80		
Widower	67 (20.55)	(−17.25)	18.35		
**Educational level**					
Primary studies	269 (8.25)	(−11.71)	19.61	<0.001	0.008 **
Secondary studies	367 (11.25)	(−13.43)	20.12		
Vocational training studies	547 (16.77)	(−11.00)	17.69		
University studies	1480 (45.38)	(−10.36)	17.98		
Post-university studies	586 (17.97)	(−7.88)	17.07		
No formal studies	12 (0.37)	(−0.75)	21.72		
**Employment status**					
Active	1891(57.99)	(−8.63)	17.96	<0.001	0.014 **
Unemployed—Home chores	606 (18.58)	(−11.96)	18.59		
Student	521 (15.98)	(−14.09)	19.21		
Retired	243 (7.45)	(−12.66)	15.61		
**Residence area**					
Urban	2271 (69.64)	(−10.69)	18.08	0.241	0.044 **
Rural	990 (30.36)	(−9.88)	18.61		
**Private access to the outside**					
Yes	2045 (62.71)	(−9.00)	17.88	0.001	0.212 *
No	1216 (37.29)	(−12.87)	18.60		
**Housing size**					
<30 m^2^	36 (1.10)	(−16.41)	21.12	0.003	0.005 **
30–60 m^2^	450 (13.80)	(−12.02)	19.39		
60–90 m^2^	1294 (39.68)	(−11.06)	18.49		
90–120 m^2^	997 (30.57)	(−9.68)	17.40		
>120 m^2^	504 (15.45)	(−8.51)	17.72		

*n*: Number of patients; OBQ: Occupational Balance Questionnaire; SD: Standard deviation; Effect size: * Hedge’s’ g, ** Partial eta squared (n^2^ *p*).

**Table 2:** Correlation between differential OBQ scores and sociodemographic variables.

	Mean	SD	r Pearson with Differential Score in OBQ	*p*-Value
Age	40.53	14.05	0.035	0.048
Days of home confinement	22.69	13.29	(−0.101)	<0.001
Number of people at home	2.96	1.23	(−0.020)	0.252
Number of children under 18 years at home	0.49	0.87	0.052	0.003
Number of dependents at home	0.18	0.51	(−0.033)	0.061
Number of rooms at home	5.27	2.17	0.021	0.232

OBQ: Occupational Balance Questionnaire; SD: Standard deviation.

**Table 3:** Comparison of differential scores in the OBQ according to COVID-19-related variables.

	*n* (%)	Differential Score in OBQ		*p*-Value	Effect Size
		Mean	SD		
**Employment status due to COVID-19**					
Stopped working—No telecommuting	798 (33.08)	(−9.70)	20.21	0.228	0.011
Working—Telecommuting	1614 (66.92)	(−9.91)	17.39		
**Telecommuting**					
Yes	1105 (45.81)	(−8.97)	17.67	0.033	0.087
No	1307 (54.19)	(−10.57)	18.91		
**Currently infected by COVID-19**					
Yes	38 (1.17)	(−18.89)	19.71	0.004	0.470
No	3223 (98.83)	(−10.34)	18.21		
**Infected by COVID-19**					
Yes	68 (2.09)	(−11.83)	20.99	0.402	0.078
No	3193 (97.91)	(−10.41)	18.18		
**Isolation**					
Yes	150 (4.60)	(−13.32)	18.69	0.048	0.165
No	3111 (95.40)	(−10.31)	18.21		
**Perception of the information received**					
Enough	1218 (37.35)	(−8.27)	16.36	<0.001	0.191
Insufficient	2043 (62.65)	(−11.74)	19.17		

*n*: Number of patients; OBQ: Occupational Balance Questionnaire; SD: Standard deviation; Effect size: Hedge´s; COVID-19: Coronavirus disease.

**Table 4:** Multiple linear regression analysis of independent predictive factors related to COVID-19 infection for a higher occupational balance.

Independent Predictive Factors	Standard Error	β	t	*p*-Value
Age	0.03	0.071	3.75	0.001
Perception of received enough information	0.78	0.071	3.34	<0.001
Being not currently infected by COVID-19	3.30	0.055	2.71	0.007
Telecommuting	0.76	0.047	−2.29	0.022

COVID-19: Coronavirus disease.
